# Short-Term Repeat Healthcare Visits and Area-Level Inequalities in a Primary Care-Centered Health System

**DOI:** 10.3390/ijerph23040410

**Published:** 2026-03-24

**Authors:** Beyza Arpaci Saylar, Bekir Aktura, Mehmet Burhan Küçükoğlu

**Affiliations:** 1 Eyüpsultan District Health Directorate, 34050 Istanbul, Türkiye; 2 Department of Public Health, Istanbul Provincial Directorate of Health, 34142 Istanbul, Türkiye; bekiraktura@gmail.com (B.A.); burhan_kucukoglu@hotmail.com (M.B.K.)

**Keywords:** primary care, repeat visits, healthcare utilization, frequent attenders, socioeconomic inequalities, SEGE index, urban health systems

## Abstract

**Highlights:**

**Public health relevance—How does this work relate to a public health issue?**

- This study addresses the growing burden of short-term repeat healthcare utilization in primary care-centered health systems, particularly in large metropolitan settings.
- It examines how socio-spatial inequalities and health system design influence early post-visit care-seeking behavior beyond individual clinical needs.

**Public health significance—Why is this work of significance to public health?**

- The findings reveal that a substantial proportion of patients generate multiple healthcare contacts within only 21 days, indicating significant system-level workload and inefficiencies.
- By integrating individual utilization data with district-level socioeconomic indicators, the study provides novel evidence on how structural inequalities shape healthcare utilization patterns.

**Public health implications—What are the key implications or messages for practitioners, policy makers and/or researchers in public health?**

- Strengthening continuity of care, care coordination, and follow-up mechanisms in primary care is essential to reduce unnecessary repeat visits and system burden.
- Policy interventions should incorporate district-level, socioeconomically targeted strategies and consider reinforcing gatekeeping and integrated care models in urban health systems.

**Abstract:**

Background: Frequent and short-term repeat visits place significant pressure on primary care-centered health systems, particularly in large metropolitan areas. Istanbul, with its high population density and heterogeneous sociodemographic profile, presents a unique context for understanding short-interval healthcare utilization dynamics. Objective: To examine short-term repeat healthcare utilization following an index primary care visit and to explore how district-level population and socioeconomic characteristics shape early post-visit care-seeking dynamics in a large metropolitan setting. Methods: This retrospective cohort study analyzed protocol records from 225 randomly selected FMUs across Istanbul. A total of 11,101 individuals who presented on 7 July 2025 were followed for 21 days, during which 26,743 healthcare contacts (index family medicine unit visits, recurrent FMU visits, and secondary/tertiary care visits) were captured. FMU repeat visits, higher-level utilization, district-level population density, and socioeconomic development level (SEGE-2022) were analyzed using descriptive statistics and district-level comparative analyses. Results: During the 21-day follow-up period, participants generated a total of 26,743 healthcare contacts. Across the full cohort, the median number of recurrent FMU visits was 0 (IQR 0–1), and the median number of secondary/tertiary visits was 0 (IQR 0–1). Among individuals who had repeat contacts, the median number of recurrent FMU visits was 1 (IQR 1–2), and the median number of secondary/tertiary visits was 1 (IQR 1–2). Repeat visits clustered in the first 7 days, whereas higher-level visits increased between days 10–21. Districts with lower SEGE status and high population density (e.g., Esenyurt, Bağcılar, Pendik, Küçükçekmece, Ümraniye) exhibited markedly higher repeat visit intensity. Spatial patterns indicated substantial clustering in western and socioeconomically disadvantaged districts. Multivariable regression analysis showed that visitor patient status was associated with higher secondary/tertiary care utilization (OR = 1.14, 95% CI 1.04–1.24), while higher SEGE scores were modestly associated with increased repeat FMU visits. Conclusions: Short-term repeat visits in Istanbul appear to be influenced not only by clinical needs but also by broader contextual factors such as socioeconomic disadvantage, population density, and health system organization. These findings suggest potential structural pressures within Türkiye’s primary care-centered system and highlight the potential value of district-specific interventions.

## 1. Introduction

Primary healthcare constitutes the fundamental cornerstone of health systems and is built upon the principles of accessibility, continuity, coordination, and comprehensive care. Evidence from various countries has demonstrated that a strong primary care structure reduces mortality and morbidity, enhances the cost-effectiveness of the health system, and contributes to diminishing health inequalities [1,2]. The World Health Organization defines primary care as the individual’s first point of contact with the health system and emphasizes strengthening this level as a key priority for achieving sustainable and universal health coverage [3]. However, limited attention has been paid to the very early period following a primary care encounter, during which patients may re-present or seek care across multiple levels of the health system.

Short-term repeat visits, bypassing of primary care toward secondary or tertiary institutions, and fragmented utilization patterns across different providers have emerged as indirect yet critical performance indicators that reflect important aspects of system functioning [4,5].

Patients within this frequent-attender group have been reported to have a higher burden of chronic disease, coexisting mental health problems, and social disadvantage; however, the continuity and coordination of the care they receive tend to be relatively weak [6]. Recurrent visits have significant implications not only for primary care services but also for emergency departments and outpatient clinics, where they have been associated with increased workload, crowding, unnecessary diagnostic tests and treatments, repeated prescriptions, and rising healthcare expenditures [5,7].

Theoretically, in health systems where primary care physicians assume a “gatekeeper” role, behaviors such as recurrent attendance are proposed to be more effectively controlled. In gatekeeping models, patients’ access to specialist and hospital services depends on the referral and guidance provided by their primary care physician [8]. Systematic reviews indicate that gatekeeping practices can reduce the use of hospital and specialist services and lower overall healthcare expenditures, while increasing the rate of initial contact in primary care and improving care coordination. However, they also show that in certain contexts, patient satisfaction and perceived accessibility may be negatively affected [8]. In Türkiye, although the family medicine model is widely implemented, there is no mandatory referral chain or strong gatekeeping function; in most cases, patients can directly access secondary and tertiary care institutions without first consulting their family physician [8,9]. This situation contributes to a fragmented and duplicative pattern of healthcare utilization, weakens continuity of care particularly among frequent attenders, and may adversely affect the overall efficiency of the health system.

Recent studies have shown that healthcare utilization behaviors cannot be explained solely by individual clinical needs or patient characteristics; rather, the socioeconomic and spatial features of the environment in which individuals live also play a substantial role in shaping these behaviors. These area-level determinants include indicators such as population density, income and education levels, employment opportunities, urban disadvantage, the distribution of health infrastructure, and physical accessibility to services [7,10,11]. This framework moves beyond individual risk factors and enables the analysis of the structural and environmental determinants of healthcare utilization patterns, thereby making socio-spatial health inequalities more visible [2,11,12].

In large metropolitan areas where socio-spatial inequalities are pronounced, these area-level determinants directly influence patterns of primary care utilization and recurrent attendance. Istanbul, with its high population density, heterogeneous socioeconomic structure, and rapid urban transformation, represents a distinctive example in this regard. The District SEGE-2022 study conducted by the Ministry of Industry and Technology reveals substantial differences in development levels across Istanbul’s districts with respect to income, education, employment, and living conditions; this indicates that even within the same provincial boundaries, access to and patterns of primary care utilization may vary considerably [13].

In Türkiye, studies on the family medicine system and healthcare utilization have predominantly focused on patient satisfaction, access, referral chain practices, or general evaluations of service delivery. Quantitative research examining recurrent attendance behavior in primary care—together with its short-term dynamics and patterns of bypassing toward higher-level institutions—within the framework of socio-spatial determinants has remained limited [4,5]. Particularly at the scale of Istanbul, the lack of large-sample studies that examine short-term repeat visits following the initial presentation to family medicine units, as well as concurrent patterns of utilization of secondary and tertiary care institutions, in relation to district-level socioeconomic indicators and population characteristics, points to a significant knowledge gap.

This study aims to analyze recurrent visits and referrals to secondary and tertiary care institutions within 21 days following the initial visit of a cohort of 11,101 individuals who presented to family medicine units in Istanbul on 7 July 2025. The study examines repeat visits to the same family medicine unit, visits to different family medicine units, and presentations to higher-level institutions, in association with district-level population size and socioeconomic development indicators. In doing so, it seeks to contribute to the evaluation of continuity and coordination of care in primary health services from a socio-spatial health inequality perspective and to provide a foundation for evidence-based policy implications regarding recurrent utilization behavior.

## 2. Methods

### 2.1. Study Design

This study is a retrospective cohort analysis of healthcare utilization patterns following an index visit to family medicine units (FMUs) in Istanbul. Individuals who presented to selected FMUs on 7 July 2025 were followed for 21 days to capture subsequent healthcare contacts across primary and higher levels of care. A 21-day follow-up period was selected to capture short-term healthcare-seeking dynamics immediately following the index primary care encounter while minimizing the influence of longer-term care trajectories.

The study aims to investigate the relationship between recurrent attendance behaviour, the utilization of higher-level healthcare institutions, spatial (district-level) variation, and socioeconomic determinants.

Data were obtained from official digital health information systems coordinated by the Istanbul Public Health Services Directorate.

### 2.2. Population and Sampling

The study population consisted of all individuals who presented to family medicine units (FMUs) across Istanbul. A multistage stratified sampling approach was employed:

1. The 39 districts of Istanbul were defined as strata.
2. District population size and the number of FMUs within each district determined stratum weights.
3. A total of 225 FMUs were selected through simple random sampling within each stratum.

Protocol records of individuals who presented to these 225 FMUs on 7 July 2025 were examined, yielding an initial cohort of 11,101 individuals. During the subsequent 21-day follow-up period, a total of 26,743 healthcare contacts (including index FMU visits, recurrent FMU visits, and secondary/tertiary care visits) were recorded.

This sampling structure produced a large cohort reflecting short-term healthcare utilization patterns across multiple districts of Istanbul. Because analytical sampling weights were not applied in the statistical models, the results should be interpreted as reflecting patterns within the sampled cohort rather than population-weighted estimates. Because the study analyzed all protocol records from the selected FMUs for the specified index date, a formal sample size calculation was not performed. The cohort therefore reflects the full available dataset within the defined sampling framework.

District population size was entered into the regression models per 100,000 population increase to improve interpretability.

### 2.3. Data Sources

Four main data sources were used:

1. Family Medicine Unit Protocol Records
  - Index visits dated 7 July 2025
  - Number of person-level visits
  - Recurrent visits to the same FMU
2. National Health System (NHS) Records
  - For the 21-day follow-up period:
  - Secondary and tertiary care visits
  - Temporal distribution of visits
  - Clinical/department-based utilization patterns
3. SEGE–2022 Socioeconomic Development Data
  - District-level socioeconomic development categories
    (district-level matching was conducted for spatial analyses)
4. Population Data
  - Updated population sizes for all 39 districts
  - Standardization of visit intensity by population size

All datasets were merged, and individual-level visit records were linked with district-level SEGE categories and population data for analysis. Because the study relied on registry-based administrative records routinely used in the national health information system, the dataset provides high completeness and minimizes recall or reporting bias. Records were chronologically ordered to ensure that follow-up contacts occurred after the index visit.

### 2.4. Variables and Definitions

#### 2.4.1. Dependent Variables

1. **Recurrent FMU Visit**

≥2 visits by the same individual to the same FMU within 21 days.

2. **Higher-Level Healthcare Utilization**

Number of visits to secondary and tertiary institutions (total and by clinical category).

#### 2.4.2. Independent Variables

- District population
- SEGE–2022 socioeconomic development level
- Visitor (non-registered patient) ratio
- Timing of visits (0–7 days, 8–14 days, 15–21 days)
- Clinical category of visit (internal medicine, pediatrics, emergency, etc.)

#### 2.4.3. Operational Definitions

- Recurrent attendance: ≥2 visits within 21 days
- High system load: Number of visits per person exceeding the citywide average
- Visitor patient: An individual seeking care at an FMU other than the one to which they are officially registered

These definitions were standardized in accordance with the literature and the structure of the dataset. For analytical purposes, multiple contacts occurring on the same day were counted as separate visits only when they were recorded as independent protocol entries representing distinct clinical encounters in the administrative database. Emergency department visits were included within the secondary/tertiary care category. Individual-level records from FMU protocol data and the national health information system were linked using anonymized patient identifiers. Data cleaning procedures included removal of duplicate records, verification of visit dates, and consistency checks across databases. No records were excluded after linkage because the administrative dataset did not contain missing identifiers required for follow-up.

### 2.5. Statistical Analysis

Data were analyzed using SPSS 26.0 (IBM Corp., Armonk, NY, USA).

**Descriptive Statistics**

- Median, minimum–maximum, interquartile range (IQR)
- Percentage distributions
- Comparative distributions at district and SEGE levels

**Analytical Approach**

The analysis focused primarily on descriptive statistics and district-level comparative patterns. Median values, interquartile ranges (IQR), and percentage distributions were calculated. Utilization patterns were examined across districts, SEGE development levels, and population size indicators. Given the absence of detailed individual-level socioeconomic variables, multivariable modelling was limited to district-level and registration-status indicators. Multivariable logistic regression models were constructed to examine factors associated with repeat FMU visits and higher-level healthcare utilization. These models were used to explore associations between area-level indicators and individual healthcare utilization outcomes. Two models were estimated. The first model examined predictors of repeat FMU visits (≥1 repeat visit within 21 days), and the second model examined predictors of secondary/tertiary care utilization. Independent variables included district population size [14], district-level socioeconomic development score (SEGE-2022), and patient registration status (registered vs. visitor patient). Odds ratios (OR) and 95% confidence intervals (CI) were calculated.

**Spatial and Socioeconomic Analyses**

- Comparison of visit intensity by district SEGE level and population size
- Map-based classification to evaluate area-level patterns

A significance level of *p* < 0.05 was considered statistically meaningful.

Because district-level socioeconomic indicators were used alongside person-level healthcare utilization outcomes, the findings should be interpreted cautiously in light of potential ecological bias. As a robustness check, alternative model specifications were examined using the same set of predictors. The direction and magnitude of the estimated associations remained consistent across models, supporting the stability of the findings.

### 2.6. Ethical Considerations

The study was conducted in accordance with the principles of the Declaration of Helsinki.

Ethical approval was obtained (Ethics Committee No: E-10840098-202.3.02-7217; Date: 20 October 2025).

All personal data were anonymized and used solely for research purposes.

### 2.7. Strengths and Methodological Contributions

This study offers several methodological strengths:

- It provides a rare large-scale area-based analysis in a megacity such as Istanbul.
- It employs a high-resolution 21-day follow-up period, capturing short-term utilization dynamics.
- It incorporates spatial matching using SEGE and population datasets.
- It simultaneously examines primary and higher-level healthcare utilization patterns.
- It contextualizes recurrent attendance behavior within social, spatial, and structural determinants.

Collectively, these features position the study as a methodologically original contribution to the literature on primary care utilization and health system performance.

## 3. Results

### 3.1. Overall Utilization Profile

A total of 11,101 index visits made to 225 family medicine units (FMUs) across Istanbul on 7 July 2025 were included in the study. During the subsequent 21-day follow-up period, these individuals generated 26,743 healthcare contacts to FMUs and secondary/tertiary care institutions. The mean number of visits per person was 2.41 (SD: 2.03; median: 2; range: 1–21).

During the follow-up period, 7347 recurrent FMU visits and 8295 secondary/tertiary care visits were recorded. Overall, FMUs accounted for approximately 69% of all healthcare contacts—including the index visit—while higher-level institutions accounted for 31%.

A total of 63.5% of the cohort (*n* = 7048) made at least one additional healthcare visit beyond the index encounter. Overall, 42.8% of individuals had at least one repeat visit to an FMU, while 41.1% had at least one secondary or tertiary care contact during the follow-up period. Approximately 20.4% of the cohort used both primary care and higher-level services after the index visit. In contrast, 36.5% of individuals had no additional healthcare contact within the 21-day observation window. Across the full cohort, the median number of recurrent FMU visits was 0 (IQR: 0–1; range: 0–11), and the median number of secondary/tertiary visits was 0 (IQR: 0–1; range: 0–13). Among individuals who made repeat visits, the median number of recurrent FMU visits was 1 (IQR: 1–2; range: 1–11) among 4750 persons, and the median number of higher-level visits was 1 (IQR: 1–2; range: 1–13) among 4561 persons.

Regarding registration status, 74.4% of the cohort were permanently registered patients, whereas 25.6% were visitor (non-registered) patients. Visitor patients accounted for 24.4% of all recurrent FMU visits and 27.1% of secondary/tertiary visits. Although the number of recurrent FMU visits per person was similar between registered and visitor patients, the average number of higher-level visits was slightly higher among visitor patients (0.73 vs. 0.79 visits/person) (Table 1).

### 3.2. Temporal Distribution of Recurrent Visits

**Figure 1:**
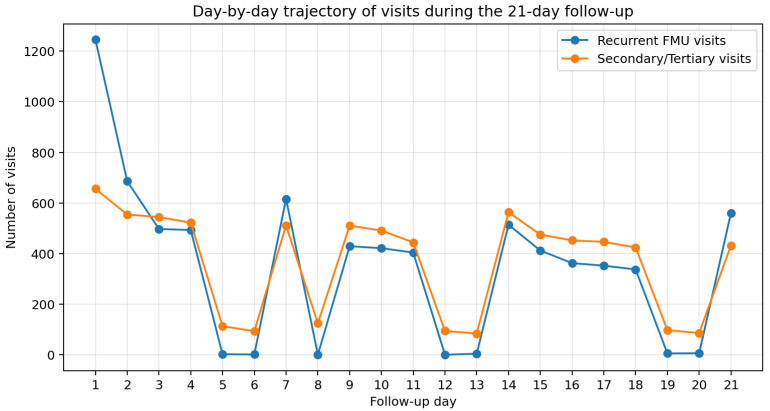
Day-by-day trajectory of recurrent family medicine unit visits and secondary/tertiary care visits during the 21-day follow-up period after the index family medicine visit.

Analysis of visit timing showed that recurrent FMU visits occurred throughout the 21-day follow-up period, with a clear concentration in the early days after the index visit. The day-by-day trajectory further demonstrated that recurrent FMU visits were most prominent during the first week, whereas secondary/tertiary care visits remained sustained across the follow-up period and became relatively more prominent in the later days (Figure 1).

Within the first 7 days (8–14 July), 3541 recurrent FMU visits were recorded, representing 48.2% of all recurrent FMU visits. In the subsequent 14 days (15–28 July), 3806 recurrent FMU visits were recorded (51.8%). Thus, repeat visits were concentrated early but persisted throughout the entire follow-up period (Figure 1).

For secondary and tertiary care visits, excluding the index day, 38.8% of the 7715 visits occurred in the first 7 days, whereas 61.2% occurred between days 8–21. This pattern indicates that higher-level care utilization remained substantial beyond the immediate post-index period and became relatively more prominent during the second and third weeks.

### 3.3. District-Level Utilization Patterns, Population Dynamics, and Visitor Patients

Index visits in the study cohort were distributed across all districts of Istanbul. Districts with the highest number of index visits included Esenyurt (*n* = 840), Bağcılar (*n* = 655), Pendik (*n* = 643), Küçükçekmece (*n* = 555), Sarıyer (*n* = 440), Üsküdar (*n* = 433), and Kartal (*n* = 406).

District-level analysis of recurrent visits demonstrated that total recurrent visit burden was concentrated particularly in high-volume districts such as Esenyurt, Bağcılar, Pendik, Küçükçekmece, and Bahçelievler. The combined recurrent visit counts (FMU + secondary/tertiary) from these five districts constituted a substantial proportion of all repeat visits.

When recurrent visit burden per capita (total recurrent visits/number of cohort individuals from each district) was examined, districts such as Bahçelievler, Beşiktaş, Sultanbeyli, Zeytinburnu, Güngören, Esenler, Bağcılar, Büyükçekmece, Kartal, and Ümraniye ranked highest. This demonstrates that recurrent attendance is not solely dependent on population size but is strongly influenced by district-level factors such as service accessibility, patient mobility, and local demographic/clinical characteristics.

According to the 2024 ADNKS population data, district populations in Istanbul range from 16,979 (Adalar) to 988,369 (Esenyurt). Esenyurt, Küçükçekmece (789,033), Pendik (749,356), Ümraniye (727,819), and Bağcılar (713,594) constitute the most populous districts [14]. These districts also appeared prominently in our cohort in terms of both absolute cohort size and total visit counts. However, per-capita recurrent visit rates were not limited to the largest districts; several medium-sized districts demonstrated similarly high rates.

Visitor patient ratios varied markedly across districts. For example, approximately 60% of patients in Beyoğlu and 50% in Bayrampaşa were visitor patients. In Zeytinburnu, Beykoz, Sarıyer, Gaziosmanpaşa, Tuzla, Esenler, Kadıköy, and Esenyurt, visitor ratios exceeded 30%. In districts with high visitor rates, total healthcare utilization reflects not only the registered resident population but also daytime population inflow and inter-district mobility.

**Figure 2:**
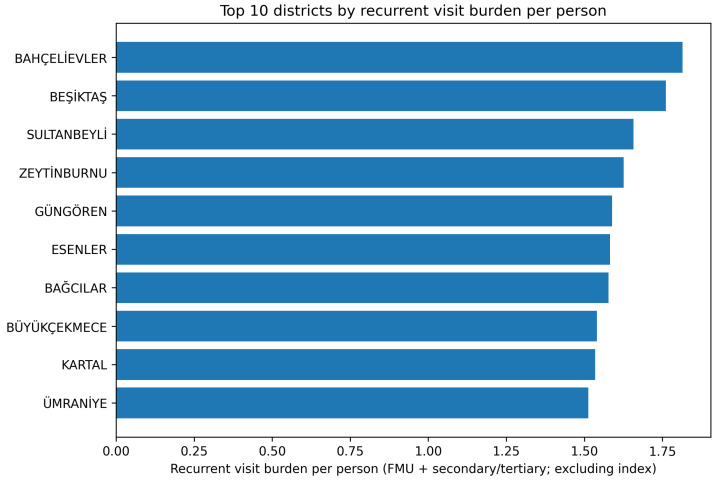
Top 10 districts by recurrent visit burden per person (recurrent FMU + secondary/tertiary; excluding index).

The SEGE-2022 study demonstrates that there are marked gradients in socioeconomic development across Istanbul’s districts and that these districts are classified into six distinct levels of development [13]. In our study, the per-capita burden of recurrent visits was high in both districts with high socioeconomic development scores (e.g., Beşiktaş, Kadıköy, Bakırköy, Bahçelievler) and in relatively disadvantaged districts (e.g., Bağcılar, Esenler, Zeytinburnu, Sultanbeyli). This pattern suggests that recurrent attendance behavior may have a non-linear and more complex relationship with socioeconomic level, and that area-level determinants should be interpreted alongside factors such as population mobility, daytime versus nighttime population differences, and the service delivery capacity of local healthcare systems (Table 2) (Figure 2).

### 3.4. Utilization of Higher-Level Care and Clinical Distribution

**Figure 3:**
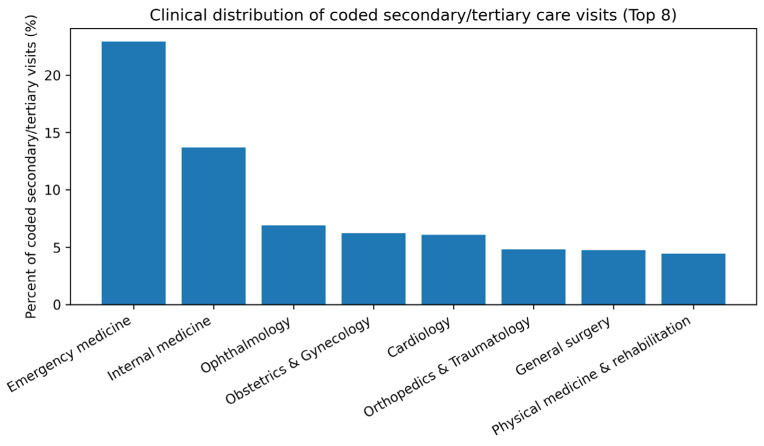
Clinical distribution of coded secondary/tertiary care visits (Top 8 departments).

Analysis of the clinical distribution of secondary and tertiary care visits showed that, among codable encounters, the most frequently visited departments were emergency medicine (21.1%), internal medicine (13.8%), and ophthalmology (7.7%). These were followed by cardiology (6.1%), obstetrics and gynecology (5.7%), orthopedics and traumatology (4.9%), general surgery (4.6%), and neurology (4.5%). Together, these eight departments accounted for more than two-thirds of all secondary/tertiary care visits (Figure 3).

This distribution suggests two distinct patterns of higher-level healthcare utilization. First, there is a substantial burden of repeat visits for acute complaints and urgent conditions (e.g., emergency medicine, internal medicine, cardiology). Second, cases requiring chronic disease management, multidisciplinary assessment, or advanced diagnostic workup (e.g., cardiology, orthopedics, neurology, physical medicine and rehabilitation) also contribute meaningfully to overall recurrent visit volume. This pattern reflects both the complexity of patient needs and potential gaps in continuity and coordination between primary and higher levels of care.

### 3.5. System Burden Assessment

Within a relatively short 21-day follow-up period, 26,743 healthcare contacts were generated from 11,101 index visits—equivalent to an average of 2.4 visits per person. This finding indicates that recurrent utilization imposes a considerable workload on the healthcare system.

This burden not only increases patient volume within FMUs but also exerts pressure on outpatient appointment capacity, emergency department crowding, waiting times, and the workload of clinical personnel in secondary and tertiary institutions. District-level and registration-status variations suggest that recurrent attendance behavior reflects a combination of individual-level factors (e.g., chronic disease burden and care needs) and area-level determinants (e.g., population density, socioeconomic structure, visitor patient ratio, service delivery capacity).

These findings highlight the need for policies aimed at strengthening continuity and coordination within primary care, improving integration with higher levels of the health system, and developing area-level targeted interventions to address structural drivers of recurrent utilization.

### 3.6. Multivariable Analysis of Repeat Healthcare Utilization

Multivariable logistic regression analysis demonstrated that visitor patient status and district-level socioeconomic conditions were associated with healthcare utilization patterns. Visitor patients had significantly higher odds of secondary or tertiary care utilization during the follow-up period (OR = 1.14, *p* = 0.003). District population size was not significantly associated with either outcome. Higher SEGE scores were associated with a modest increase in repeat FMU visits (OR = 1.05, *p* = 0.013), indicating that recurrent primary care utilization may also occur in socioeconomically developed districts (Table 3). Overall, the regression models demonstrated modest effect sizes, suggesting that recurrent healthcare utilization is likely shaped by multiple interacting determinants rather than a single dominant factor.

## 4. Discussion

This study provides a high-resolution analysis of recurrent healthcare utilization by examining 26,743 healthcare contacts made within 21 days following the index presentations of 11,101 individuals to family medicine units (FMUs) in Istanbul. The findings show that a substantial volume of repeat visits occurs even within a short timeframe. This pattern does not appear random but reflects a systematic behavior shaped by the interaction of individual needs, health system design, socioeconomic inequalities, and spatial factors. Rather than estimating long-term utilization burden, these findings should be interpreted as an early signal of potential challenges in care continuity and coordination following an initial primary care encounter. These findings may therefore be interpreted not only as utilization statistics but also as an early signal of structural pressures within a metropolitan primary care system.

**Interpretation of Key Findings and Healthcare Utilization Behavior**

Across the full cohort, the median number of recurrent FMU visits and secondary/tertiary visits was zero. Among individuals who made repeat contacts, the median number of recurrent FMU visits was 1 and the median number of secondary/tertiary visits was also 1. The concentration of FMU visits within the first 7 days, contrasted with the clustering of hospital visits between days 10–21, suggests a pattern in which some patients seek additional care after the initial encounter. The international literature similarly reports that short-term repeat visits are frequently associated with weak continuity of care, unclear follow-up plans, and patients’ perceptions of elevated clinical risk [4,5,15]. These patterns may be interpreted in light of the literature on continuity of care, uncertainty, and metropolitan service organization.

**The Andersen Model and the Context of a Free-Access Health System**

When recurrent attendance behavior is examined through the lens of Andersen’s Behavioral Model of Health Services Use, it becomes evident that predisposing factors (e.g., age, perceived health status), enabling factors (e.g., access regulations, transportation, the right to direct access without referral), and need factors operate simultaneously in shaping this pattern of utilization [16,17,18]. The absence of a mandatory referral chain in Türkiye and the widespread availability of direct access to higher-level institutions create an environment in which enabling factors become dominant. Such patterns can weaken continuity of care, lead to unnecessary repetition of diagnostic tests, and generate information gaps that may compromise patient safety. The findings indicate that recurrent attendance may partly reflect characteristics of the health system design rather than solely individual patient preferences.

The regression analysis further supports the role of patient mobility within metropolitan healthcare systems. Visitor patients were more likely to utilize secondary or tertiary healthcare services, suggesting that mobility and care fragmentation may contribute to higher-level service utilization.

**International Comparison: The Frequent Attender Literature**

In many countries, the “frequent attender” group constitutes only a small proportion of the patient population, yet it accounts for a disproportionately large share of total healthcare visits, costs, and emergency department burden [5,9,15,19]. In England, the Netherlands, and Scandinavian countries, a threshold of ≥12 visits per year is commonly used to define frequent attenders, whereas in Istanbul, utilization levels approaching this threshold were observed within only 21 days—highlighting the exceptionally high intensity of service use in this setting. Similarly, data from Taiwan, Republic of Korea, and Canada have shown that the annual healthcare costs of frequent attenders are two to three times higher than those of other users [9,15,19].

**Socioeconomic inequalities and Area-level determinants**

The pronounced concentration of recurrent visits in districts with lower SEGE-2022 socioeconomic levels and higher populations indicates that spatial inequalities are a powerful determinant of healthcare utilization. International studies similarly demonstrate that individuals residing in disadvantaged neighborhoods tend to exhibit more irregular, repetitive, and multi-institutional care-seeking patterns [2,11,12]. These findings suggest that recurrent attendance may be influenced not only by clinical factors but also by social gradients and spatial inequalities.

Interestingly, the regression analysis indicated that higher SEGE scores were modestly associated with increased repeat FMU utilization. This finding suggests that recurrent primary care visits may not be confined to socioeconomically disadvantaged districts but may also occur in highly developed urban areas where service accessibility and patient mobility are high.

**System and Policy-Level Implications**

The findings of this study highlight the need for multilevel interventions to reduce recurrent attendance behavior:

Strengthening information continuity: Standardizing bidirectional feedback and decision-support mechanisms between FMUs, hospitals, and private-sector information systems may reduce repeat visits driven by uncertainty and fragmented information flow.

Regional capacity planning: Monitoring district-level visit burden and visitor-patient flows can inform workload balancing across teams; in high-burden districts, solutions such as task-sharing, remote monitoring, and rapid-access appointment slots should be implemented.

Reinforcing the gatekeeping function: Even without introducing a mandatory referral chain, strengthening clinical pathways and follow-up protocols may help rationalize referrals to secondary and tertiary levels of care [7,10,20].

**Strengths and Limitations**

This study is based on a large multi-district dataset derived from 225 FMUs across Istanbul and is among the few investigations to examine short-term healthcare utilization at high resolution. The use of registry-based data minimizes respondent bias. However, the relatively short follow-up period may not fully capture long-term behavioral patterns, and the limited availability of individual-level sociodemographic variables constrains causal inference. In addition, clinical diagnoses, symptom severity, and comorbidity profiles were not available in the administrative dataset, which limits the interpretation of the clinical drivers of repeat visits. Furthermore, the analysis did not incorporate individual-level socioeconomic variables, and therefore the observed associations with district-level indicators should be interpreted as contextual rather than causal.

Another limitation is the relatively short follow-up period of 21 days. Although this timeframe allows for the identification of early repeat utilization patterns after the index visit, it may not fully capture longer-term healthcare-seeking trajectories or persistent frequent attendance patterns.

In addition, the administrative dataset did not include individual-level socioeconomic or clinical variables such as age, sex, comorbidities, or disease severity; therefore, the analysis relied partly on district-level indicators, and the observed associations should be interpreted with caution due to the potential for ecological bias and the inability to adjust for patient-level confounding factors.

**Future Research**

Future studies should incorporate measures of continuity of care, symptom categories, comorbidities, chronic disease burden, and coordination of care. Employing multilevel statistical models is recommended to further elucidate the relative influence of area-level determinants. Additionally, the effectiveness of digital feedback tools, risk-based recall systems, and primary care-centered integrated care models should be evaluated through prospective research.

## 5. Conclusions and Recommendations

### 5.1. Conclusions

This study demonstrates that the 11,101 individuals who presented to family medicine units (FMUs) in Istanbul generated 26,743 healthcare contacts within only 21 days, revealing a strikingly high intensity of utilization and clear patterns of recurrent attendance over a short period. The findings indicate that recurrent attendance is not random; rather, it represents a predictable and systematic pattern of healthcare use, concentrated within specific patient groups.

The results suggest that recurrent attendance may be influenced not solely by clinical need but also by a multilayered set of determinants—including health system design (notably the absence of a mandatory referral chain), socioeconomic conditions, spatial inequalities, and urban population density.

The higher frequency of recurrent visits observed in districts with lower SEGE-2022 socioeconomic levels and larger populations (e.g., Esenyurt, Bağcılar, Küçükçekmece, Pendik, Ümraniye) demonstrates that healthcare-seeking behavior appears to be influenced by socio-spatial inequities. Conversely, more regular utilization patterns in higher-SEGE districts suggest that health system navigation and care coordination vary systematically along the social gradient.

Across the full cohort, median recurrent FMU visits and secondary/tertiary visits were zero; among individuals with repeat contacts, the median number of visits was 1 for both categories. These patterns may reflect challenges in follow-up planning and continuity of care within densely populated metropolitan settings. The increased frequency of higher-level visits during days 10–21 further suggests a shift driven by persistent symptoms, diagnostic uncertainty, or patient preferences for hospital-level evaluation.

Taken together, the recurrent attendance patterns observed in Istanbul may serve as an early warning signal regarding health system sustainability, resource utilization, continuity of care, and socioeconomic inequalities.

Multivariable analysis further highlighted the influence of visitor patient status on higher-level healthcare utilization patterns.

This study is among the few analyses in Türkiye to examine short-term recurrent primary care utilization through spatial, socioeconomic, and system-design perspectives. It provides a robust evidence base to inform ongoing debates on health policy, primary care strengthening, and equitable service delivery.

Monitoring short-term recurrent utilization patterns may provide an important early indicator for evaluating the resilience, coordination capacity, and equity of primary care systems in large metropolitan settings.

### 5.2. Recommendations

**Early Identification of High-Risk Recurrent Attender Groups**

Risk stratification algorithms should be developed to identify individuals who make multiple visits within a 21-day period.

Multidisciplinary case-management models—integrating family physicians, nursing services, psychosocial support, and, when necessary, social work—should be implemented for these patients.

**Strengthening the Coordination Role of Primary Care**

The role of family physicians should be reinforced not only as the first point of contact but also as coordinators of care.

Chronic disease management, counseling, follow-up services, and home-based care should be expanded and supported.

Feedback loops between primary care and higher-level institutions should be standardized.

**Rational and Graduated Gatekeeping Model**

A non-coercive, guideline-based gatekeeping model monitored through quality indicators should be established.

Such a model has the potential to reduce unnecessary referrals to higher levels of care and strengthen continuity of care [8].

**District-Level Intervention Packages with a Strong Socioeconomic Focus**

In districts with low SEGE levels and high population density:

The population per primary care unit should be reduced.

Team structures should be strengthened by incorporating nurse, midwife, and psychologist support.

Dedicated follow-up programs for chronic disease management and vulnerable groups should be implemented.

District-level monitoring of recurrent attendance should be used to develop targeted intervention plans for high-risk areas.

**Recommendations for Future Research**

Longer follow-up periods should be used to examine the persistence and longitudinal trajectories of recurrent attendance patterns.

Multilevel models incorporating individual-level socioeconomic data are needed to better disentangle the relative contributions of personal and area-level determinants.

Conducting similar cohort studies at regular intervals in Istanbul will be critical for evaluating the impact of policy changes and system-level interventions over time.

### 5.3. Key Takeaway Message

This study clearly demonstrates that recurrent attendance in Istanbul is not merely a reflection of individual healthcare needs; rather, it is a structural phenomenon emerging at the intersection of socioeconomic disadvantage, spatial inequalities, and health system design.

The pattern—generating 26,743 healthcare contacts in just 21 days—may serve as an early warning signal, underscoring the need to reconsider the capacity, integration, and coordination strength of primary care.

Recurrent attendance is a multidimensional health system issue that requires comprehensive solutions—not solely individual-level interventions, but structural reforms encompassing system design, social policy, and spatial planning.

## Figures and Tables

**Table 1:** Overall healthcare utilization characteristics of the study cohort (derived from dataset).

Variable	Value
Number of individuals (index FMU visits)	11,101
Recurrent FMU visits (8–28 July)	7347
Secondary/tertiary visits (7–28 July)	8295
Total healthcare contacts captured in dataset (index + recurrent FMU + secondary/tertiary)	26,743
Mean visits per person	2.41
Median visits per person (IQR)	2 (1–3)
Registered patients, *n* (%)	8258 (74.4%)
Visitor patients, *n* (%)	2843 (25.6%)

**Table 2:** Districts with the highest and lowest recurrent visit burden per person (FMU + secondary/tertiary; excluding index).

Group	District	Cohort *n*	Recurrent Total	Burden/Person	Recurrent FMU	Secondary/Tertiary	Visitor %
Top 10	BAHÇELİEVLER	382	693	1.814	318	375	27.0
Top 10	BEŞİKTAŞ	134	236	1.761	124	112	11.9
Top 10	SULTANBEYLİ	213	353	1.657	140	213	30.0
Top 10	ZEYTİNBURNU	155	252	1.626	113	139	39.4
Top 10	GÜNGÖREN	175	278	1.589	128	150	29.7
Top 10	ESENLER	335	530	1.582	232	298	33.4
Top 10	BAĞCILAR	655	1033	1.577	518	515	22.9
Top 10	BÜYÜKÇEKMECE	263	405	1.54	195	210	9.5
Top 10	KARTAL	406	623	1.534	331	292	22.2
Top 10	ÜMRANİYE	273	413	1.513	208	205	23.1
Bottom 10	MALTEPE	373	380	1.019	173	207	1.6
Bottom 10	ARNAVUTKÖY	322	352	1.093	160	192	21.1
Bottom 10	BEYKOZ	273	319	1.168	124	195	37.4
Bottom 10	BAYRAMPAŞA	289	342	1.183	164	178	49.5
Bottom 10	FATİH	356	427	1.199	214	213	28.7
Bottom 10	ESENYURT	840	1062	1.264	487	575	32.6
Bottom 10	BEYLİKDÜZÜ	291	369	1.268	172	197	4.1
Bottom 10	KÜÇÜKÇEKMECE	555	731	1.317	366	365	28.1
Bottom 10	ŞİŞLİ	80	106	1.325	54	52	5.0
Bottom 10	AVCILAR	305	411	1.348	212	199	8.2

**Table 3:** Multivariable logistic regression analysis of healthcare utilization.

Predictor	Repeat FMU Visit OR (95% CI)	*p*-Value	Higher-Level Visit OR (95% CI)	*p*-Value
Visitor patient	0.97 (0.89–1.06)	0.55	1.14 (1.04–1.24)	0.003
District population (per 100,000 increase)	0.996 (0.98–1.01)	0.67	1.005 (0.99–1.02)	0.62
SEGE score	1.05 (1.01–1.09)	0.013	0.97 (0.94–1.01)	0.11

OR: odds ratio; CI: confidence interval. Visitor patient refers to individuals who received care from a family physician to whom they were not formally registered. SEGE score represents district-level socioeconomic development according to the SEGE-2022 index. District population represents the district population size entered into the model per 100,000 population increase.

## Data Availability

The datasets analyzed during the current study are not publicly available due to institutional data protection regulations and privacy restrictions but are available from the corresponding author on reasonable request and with permission of the Istanbul Provincial Health Directorate.
